# Metabolic Syndrome-Associated Pulmonary Lipid and Iron Accumulation and Alterations in TLR4/NF-κB Signaling and Ferroptosis-Regulatory Proteins Are Attenuated by Resveratrol Plus Quercetin in Rats

**DOI:** 10.3390/cimb48090913

**Published:** 2026-09-05

**Authors:** María Esther Rubio-Ruíz, Agustina Cano-Martínez, Jimena Alejandra Méndez-Castro, Itzel Yoandra Varona-Yañez, Elizabeth Carreón-Torres, Eulises Díaz-Díaz, María del Pilar Ramos-Godinez, Criselda Mendoza-Milla, Alfredo Cruz-Gregorio

**Affiliations:** 1 Department of Physiology, Instituto Nacional de Cardiología Ignacio Chávez, Juan Badiano 1, Sección XVI, Tlalpan, Mexico City 14080, Mexico; esther.rubio@cardiologia.org.mx (M.E.R.-R.); agustina.cano@cardiologia.org.mx (A.C.-M.); mndzc.alejandra@gmail.com (J.A.M.-C.); yoandra@ciencias.unam.mx (I.Y.V.-Y.); 2 Department of Molecular Biology, Instituto Nacional de Cardiología Ignacio Chávez, Juan Badiano 1, Sección XVI, Tlalpan, Mexico City 14080, Mexico; elizact73@gmail.com; 3 Department of Reproductive Biology, Instituto Nacional de Ciencias Médicas y Nutrición Salvador Zubirán, Vasco de Quiroga 15, Sección XVI, Tlalpan, Mexico City 14000, Mexico; eulisesd@yahoo.com; 4 Department of Electron Microscopy, Instituto Nacional de Cancerología, San Fernando No. 22, Sección XVI, Tlalpan, Mexico City 14080, Mexico; mramosg@incan.edu.mx; 5 Research Unit, Signal Transduction Laboratory, Instituto Nacional de Enfermedades Respiratorias Ismael Cosío Villegas, Calzada de Tlalpan 4502, Belisario Domínguez Sección XVI, Tlalpan, Mexico City 14080, Mexico; criselda.mendoza@iner.gob.mx

**Keywords:** metabolic syndrome, ectopic fat deposition, ferroptosis-related protein expression, inflammation, lung injury, resveratrol, quercetin, TLR4

## Abstract

Metabolic syndrome (MetS) is associated with ectopic fat deposition, chronic systemic inflammation, oxidative stress, and increased susceptibility to organ dysfunction. While its cardiovascular consequences have been extensively studied, pulmonary al-terations remain less well characterized, particularly with respect to ferropto-sis-regulatory pathways. In this study, we evaluated ectopic fat deposition, TLR4/NF-κB signaling pathway, and ferroptosis-regulatory markers in a MetS rat model and analyzed the therapeutic potential of combined resveratrol plus quercetin (combined R + Q) supplementation. Rats were maintained for five months with 30% sucrose in drinking water to induce MetS and subsequently treated for 4 weeks with the R + Q combination (50 and 0.95 mg/kg/day, respectively). Lung tissue was analyzed by fluorescence microscopy to assess fat deposition, by colorimetric histology to evaluate hemosiderin-containing cells, including the quantification of hemosiderin-laden macrophages (HLMs), and by immunoblotting to determine TLR4, p-p65, and glutathione peroxidase 4 (GPX4), as well as cystine/glutamate antiporter SLC7A11 (xCT) protein expression. Lungs from MetS rats exhibited significantly higher fat deposits, pathological HLMs accumulation, upregulated TLR4/p-p65 signaling, elevated compensatory GPX4 expression, and depressed xCT levels compared with controls. Notably, the combined R + Q treatment markedly attenuated alterations in TLR4/NF-κB signaling and successfully restored xCT expression (*p* = 0.0116), stabilizing pulmonary redox homeostasis and limiting overall tissue susceptibility to lipotoxic injury.

## 1. Introduction

Metabolic syndrome (MetS) is a multifactorial disorder characterized by central obesity, insulin resistance (IR), dyslipidemia, and chronic low-grade inflammation, markedly increasing cardiometabolic risk worldwide [1]. Although its deleterious effects on liver and cardiovascular tissues are well established, growing clinical and experimental evidence indicates that MetS also impacts pulmonary structure and function [2,3,4]. Patients with obesity and MetS exhibit impaired lung mechanics, increased airway resistance, and heightened inflammatory responses [2].

Despite growing evidence that obesity and metabolic dysfunction are associated with pulmonary alterations, the mechanisms underlying metabolic syndrome-associated changes within lung tissue remain incompletely understood [4,5]. In particular, the potential interplay among pulmonary lipid accumulation, inflammatory signaling, iron/hemosiderin deposition, and alterations in ferroptosis-regulatory pathways has not been fully elucidated. Ectopic lipid deposition is a hallmark of MetS and an important contributor to lipotoxic alterations in multiple organs [6]. While hepatic steatosis and myocardial lipid overload have been extensively characterized, less is known about the mechanisms underlying lipid accumulation and remodeling within lung tissue in the context of MetS. The lung relies on tightly regulated lipid metabolism, particularly for surfactant synthesis and homeostasis, while pulmonary cells can also utilize fatty acids and other metabolic substrates to meet their energetic demands [7,8,9]. Therefore, excessive or dysregulated lipid accumulation may reflect metabolic disturbances that could contribute to altered pulmonary homeostasis and inflammatory responses [10].

Moreover, emerging studies indicate that obesity and IR, key features of MetS, are associated with altered surfactant composition, increased lipid droplet formation in alveolar epithelial cells, and enhanced inflammatory signaling within lung tissue [11,12]. Lipid overload increases the abundance of polyunsaturated fatty acids in cellular membranes, potentially rendering tissues more susceptible to oxidative damage through lipid peroxidation [13]. Several mechanisms have been proposed to explain the association between lipid overload and inflammation; however, the molecular pathways underlying MetS-associated pulmonary alterations remain incompletely defined [2,14]. In this aspect, saturated fatty acids and oxidized lipids have been reported to activate Toll-like receptors (TLRs), particularly TLR4, a pattern-recognition receptor that can activate the nuclear factor kappa B (NF-κB) signaling pathway, thereby promoting the transcription of proinflammatory mediators and contributing to inflammatory processes in several lung diseases [15,16].

On the other hand, iron metabolism is increasingly recognized as a central regulator of metabolic inflammation and redox homeostasis. Obesity and MetS are associated with altered systemic iron handling, hepcidin dysregulation, and tissue-specific iron redistribution [17,18]. Excess redox-active iron promotes the generation of reactive oxygen species via Fenton chemistry, thereby amplifying oxidative stress, particularly in lipid-rich environments [19]. In the lung, altered iron homeostasis and iron accumulation have been associated with oxidative stress, inflammatory activation, and structural remodeling [20,21].

The convergence of lipid overload and iron dysregulation establishes a biochemical framework conducive to ferroptosis, a regulated cell death modality driven by iron-dependent accumulation of lipid peroxides [22]. Ferroptosis is tightly controlled by the glutathione peroxidase 4 (GPX4)/glutathione axis, which detoxifies phospholipid hydroperoxides and preserves membrane integrity [23]. Increasing evidence implicates ferroptosis in metabolic diseases, including obesity-related organ dysfunction and chronic inflammatory states [24,25]. Moreover, recent studies have identified ferroptosis as a contributor to pulmonary pathologies, including acute lung injury, chronic obstructive pulmonary disease, and fibrotic remodeling [26,27]. However, whether MetS promotes a ferroptosis-regulatory protein phenotype in lung tissue remains unclear.

Natural polyphenols such as resveratrol (R) and quercetin (Q) possess antioxidant, anti-inflammatory, and iron-modulating properties. Notably, recent studies suggest that polyphenols can modulate ferroptosis-related pathways through effects on lipid metabolism, redox balance, and iron handling [28,29,30]. In pulmonary models, R and Q attenuate oxidative stress and inflammatory responses [31,32]. Nevertheless, their capacity to modulate the interplay among lipid metabolism, iron handling, and ferroptosis-related regulatory pathways in the lung under chronic metabolic stress remains poorly understood.

Therefore, the aim of the present study was to determine whether sucrose-induced MetS promotes pulmonary lipid accumulation, alterations in TLR4/NF-κB signaling, iron deposition, and remodeling of ferroptosis-regulatory pathways, and to evaluate whether administration of the combined R + Q mix could regulate these processes that could cause pulmonary metabolic remodeling/alterations.

## 2. Materials and Methods

### 2.1. Animals and Experimental Design

**Figure 1:**
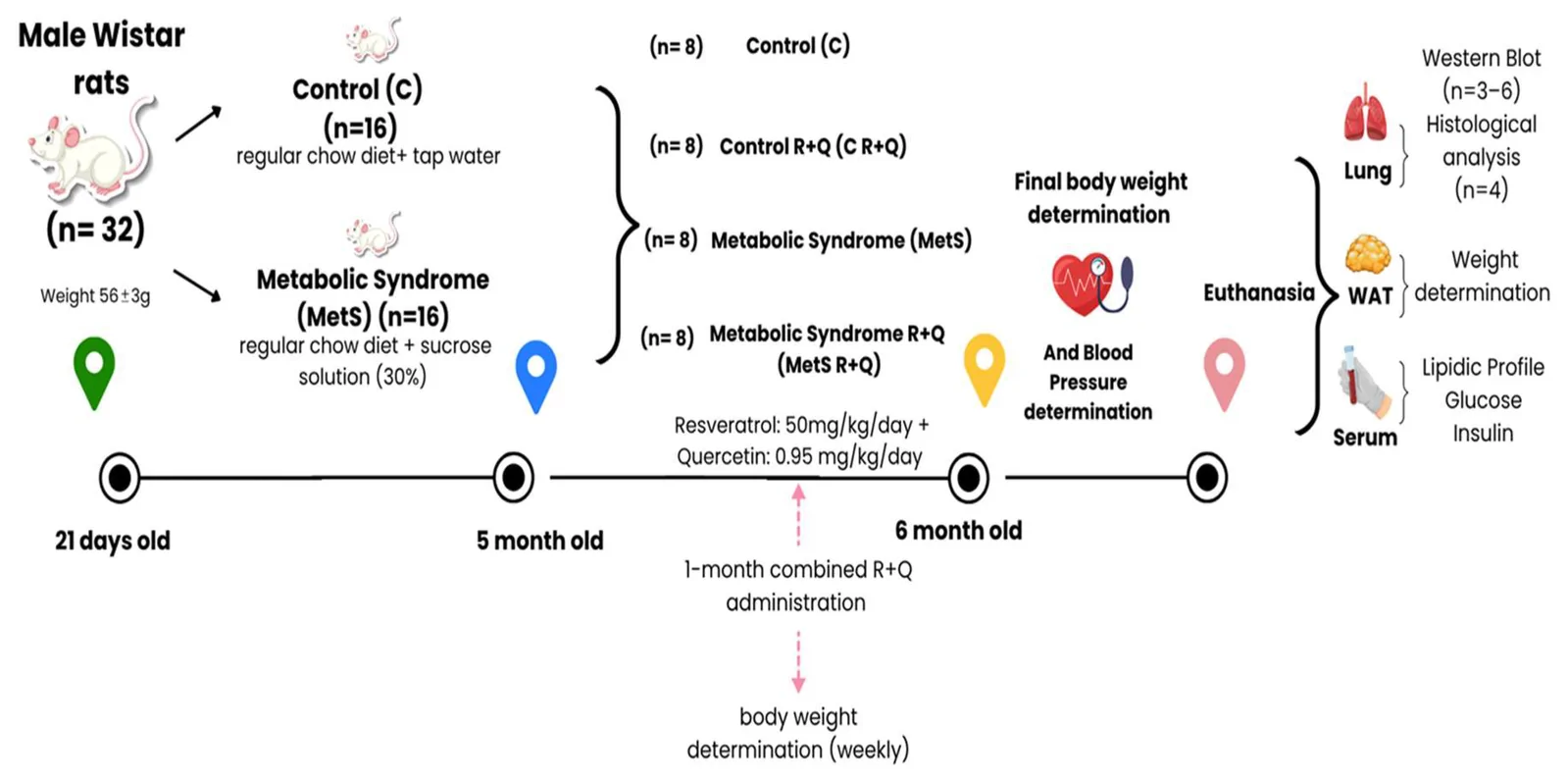
Experimental workflow and sample processing schematic. Chronological timeline of the study design highlighting initial animal group allocation (*n* = 8 per group), sequential experimental phases, and subsequent tissue processing.

Newly weaned 32 male Wistar rats (21 days old, weight 56 ± 3 g) were provided by the Institute’s animal facility (National Institute of Cardiology Ignacio Chávez) as part of research protocol #22-1342, which received institutional approval from the ethics committee. They were fed ad libitum with the regular chow diet (LabDiet 5001, PMI Nutrition International, LLC., Brentwood, MO, USA). Two groups were formed, Control (C) and MetS, by randomly selecting 16 rats each. Two animals were placed per box (8 boxes/group) (polycarbonate (480 mm × 375 mm × 210 mm, Eurostandard type IV) with autoclaved wood shavings (from Bioinvert, EDOMEX, Mexico) as bedding material and with a polycarbonate tunnel (burrow simulator; as part of environmental enrichment). During the first five months, the animals in the Control (C) group (*n* = 16) drank tap water, and the MetS group (*n* = 16) received 30% sucrose in their drinking water. In the sixth month, both cohorts were subdivided to establish the four final experimental groups (*n* = 8 per group): Control + vehicle, Control + combined R + Q, MetS + vehicle, and MetS + combined R + Q. The treatment was administered strictly through voluntary drinking water or sucrose solution, and not by oral gavage, daily for 4 weeks at a target dose of 50 mg/kg/day R + 0.95 mg/kg/day Q (provided by ResVitalé™, Boca Raton, FL, USA; which contains 20 mg of Q per 1050 mg of R). This specific mixture was selected over individual compounds to model the biological effects of commercially available nutraceutical formulations, aiming to elucidate the physiological mechanisms that remain poorly understood. The administered dose was established based on a thorough literature review identifying concentrations with proven metabolic efficacy in rodent models. Furthermore, this specific dosage was selected in accordance with our previous investigations, which demonstrated optimized homeostatic and metabolic outcomes compared with alternative concentrations of the mixture [30,33,34]. We first dissolved the natural compounds in 1 mL of a 20% ethanol solution, then added this mixture to the volume of water or sucrose solution required for each animal. The amount of water or sucrose solution the rats drank was measured daily, and the concentration of the treatment was adjusted weekly based on the 7-day average of fluid ingestion and the weekly body weight of each animal to guarantee strict adherence to the calculated target dose. Groups C and MetS without the combined R + Q treatment received only the vehicle (1 mL of 20% ethanolic solution) in tap water or in sucrose solution, respectively. Body weights of the rats were recorded weekly, and at the end of the 4-week treatment period, the systolic blood pressure was measured using the tail-cuff technique [33]. The complete overview of the treatment groups, initial sample sizes, intervention duration, vehicle compositions, and sequential endpoint tissue analyses is visually detailed in our experimental workflow schematic (Figure 1).

### 2.2. Randomization and Blinding Protocol

A stratified, two-stage randomization procedure and a strict separation of duties protocol were employed to ensure baseline homogeneity and eliminate observer bias. Initially, the 32 rats were divided into the Control group and the MetS-induction group. Prior to the natural compound treatment, animals within each main category were ranked according to their body weight and baseline metabolic parameters. Then, they were allocated to their respective subsequent sub-groups (with or without R + Q treatment) using a randomized block design. This two-stage allocation process guaranteed that baseline metabolic severity was balanced and statistically equivalent across all experimental conditions before treatments were initiated.

### 2.3. Euthanasia, Tissue Collection, and Processing

The day before euthanasia, food was withheld. Euthanasia was performed by decapitation, and blood was collected. Euthanized animals were placed on a dissection tray in a dorsal recumbent position, and under aseptic conditions, the abdominal cavity was exposed, followed by the thoracic cavity, removal of the diaphragm, subsequent rib sectioning, and the entire heart–lung units were collected. Following euthanasia, intra-abdominal white adipose tissue was carefully dissected and harvested using surgical scissors. The wet weight of the tissue was immediately recorded using a precision balance, after which the samples were properly disposed of. Lung tissue was separated from the heart over a glucose PBS solution [195 mM], and the accessory lung lobe was subsequently extracted. After removing blood residues, the lung tissue was fixed in 4% PFA for 1 week at 4 °C.

### 2.4. Serum Biochemistry Parameters

The blood sample was collected from fasting rats (12 h) and then centrifuged for 20 min at 644 rcf, at room temperature (RT). The serum was separated and stored at −70 °C until the analysis of blood chemistry was performed. Serum glucose, total cholesterol, and triglycerides were measured by enzymatic colorimetric methods using reagents from Pointe Scientific (Pointe Scientific Inc., Canton, MI, USA) and an automated biochemical analyzer Mindray BS-200 (Mindray Medical International Limited, Shenzhen, China). High-density lipoprotein (HDL) cholesterol and non-HDL-c concentrations were determined as previously described [28]. Serum concentrations of insulin were measured using a radioimmunoassay (RIA) developed and validated in the “Salvador Zubirán” National Institute of Medical Sciences and Nutrition, Mexico City; the same as those previously described [35].

### 2.5. Histological Sectioning (Slide Preparation)

Once fixed, lung tissue was washed with PBS (5 × 3 min) with moderate agitation at RT and then deposited in sucrose (30% in PBS, 24–48 h, 4 °C). After removing excess sucrose, each tissue section was coated with OCT (Optimal Cutting Temperature, Sakura Finetek Inc. Torrance, CA, USA) and frozen at −18–20 °C for cross-sectioning (10 μm) by freezing in a cryostat [Minotome Plus (Triagle Biomedical Science), Durham, NC, USA]. Tissue sections from four different animals in each group were mounted on an OCT block, with at least two blocks per slide (*n* = 4 × 2/slide), and kept at 4 °C until their subsequent use. In one set of slides, neutral fats (triglycerides) were detected using the Oil Red (OR) assay. In another set of slides, non-heme iron deposits (hemosiderin) and iron salts were detected by Perls’ Prussian blue (PPB) staining, as indicators of pulmonary iron/hemosiderin deposition and were interpreted in the context of alterations in ferroptosis-regulatory proteins.

### 2.6. Neutral Fat (Triglyceride) Detection: Oil Red (OR) Assay

Once the tissue on the slides was dried for 30 min at RT, it was fixed for 90 min at RT with calcium-buffered formalin. Three washes (5 min each) were performed with deionized water, followed by immersion in a saturated 0.5% OR (#00625 from Sigma-Aldrich, St Louis, MO, USA) solution in isopropyl alcohol for 30 min, and then three washes (30 s each) with deionized water. The slides were mounted with mounting medium (Vectashield H-100, Vector Laboratories, Inc. Burlinggame, CA, USA) for fluorescence microscopy evaluation [36].

### 2.7. Detection of Hemosiderin and Iron Salts: Perls’ Prussian Blue (PPB) Staining

One slide from each condition was selected and dried at RT for 30 min. Following the supplier’s instructions [Perls’ Staining Kit (HYCEL-Cat. 64320, Hycel de México, S.A. de C.V., Zapopan, JAL, México)]. The slides with the histological sections were placed in a freshly prepared mixture of equal parts reagent 1 (potassium ferrocyanide) and reagent 2 (5% hydrochloric acid) and incubated for 30 min at RT. After this time, they were rinsed with distilled water and exposed to nuclear red stain (5 min). The excess nuclear red was removed by washing twice in distilled water, followed by clearing in ethyl alcohol (OH) (96% OH, 1 min, twice; absolute OH, 1 min, twice) and xylene (1 min, twice), and finally mounted with synthetic resin [Entellan, catalog number 14800, Merck KGaA, DA, Germany; distributed by Electron Microscopy Sciences (EMS), Hatfield, PA, USA].

### 2.8. Image Acquisition and Quantification

Oil Red (OR) staining analysis was performed using fluorescence microscopy on a Floid Cell Imaging Station (Life Technologies, Waltham, MA, USA) to enhance signal-to-noise ratios and improve quantitative precision. Neutral fat deposition was evaluated by quantifying the Integrated Optical Density (IOD) from at least four independent tissue images (20×) per animal, across four different animals per group (yielding a minimum of 16 images per group). Image processing, spatial calibration, background correction, and automated densitometric measurements were carried out using Image-Pro Premier software version 9.0 (Media Cybernetics, Rockville, MD, USA). Background correction was achieved by establishing an auto-fluorescence threshold in non-tissue areas, which was subsequently subtracted from the raw IOD measurements. Lipid accumulation was expressed as the mean red intensity per unit area (lum/pix^2^), representing the spatially calibrated luminance per square pixel within the designated region of interest [37]. This automated approach ensured objective, high-throughput quantification while minimizing potential observer bias. PPB^+^ staining examination was performed on an Olympus BX51 microscope (Olympus, Hachioji, TYO, Japan), and images (4×) were acquired using a Q-Imaging camera (MicroPublisher 5.0, REUZEit, Temecula, CA, USA) connected to Image-Pro Plus software (version 9.0, Media Cybernetics, Rockville, MD, USA). To reconstruct each whole lung section, two complete tissue sections were scanned per animal (*n* = 4 animals per group) by manually stitching multiple sequential 4× fields (averaging 16 ± 6 fields per section). Quantitative evaluation was performed across these fully reconstructed whole lung sections to measure the total tissue area and count the number of PPB^+^ (blue) cells. The final ratio (number of PPB^+^ cells/mm^2^) was calculated for each section and subsequently averaged within each individual animal prior to statistical analysis. This workflow ensured that the individual animal remained the true biological statistical unit, preventing spatial sampling bias and eliminating the risk of pseudoreplication.

### 2.9. Western Blotting Analysis of the Levels of TLR4/NF-κB and Ferroptosis-Related Proteins

The tissues were homogenized using a porcelain mortar, chilled in liquid nitrogen, and ground; then 500 μL of lysis buffer with Thermo Scientific™ Halt™ Protease & Phosphatase Inhibitor Single-use Cocktail, EDTA-free (REF 1861280, LOT WE319345) (from Thermo Scientific, Rockford, IL, USA) was added and homogenized with a Teflon pestle. The homogenate was centrifuged at 19,954× *g* for 15 min at 4 °C; the supernatant was collected and stored at −70 °C. The protein concentration of each sample was assessed using the Bradford method [38]. Fifty micrograms of protein were isolated via SDS-PAGE and transferred to a PVDF membrane (Millipore Corporation, Billerica, MA, USA). Prior to primary antibody incubation, the membranes were blocked with 5% non-fat dry milk (Santa Cruz Biotechnology, Santa Cruz, CA, USA) in TBS-Tween. The primary antibodies used were: cystine/glutamate antiporter SLC7A11 (xCT) and GPX4 (#12691 and #52455, respectively, from Cell Signaling Technology, Danvers, MA, USA), TLR4 and pSer536-p65 NF-κB (ab217274 and ab28856, respectively, from Abcam PLC, Cambridge, Cambs, UK), which were diluted at a 1:1000 ratio in TBS-Tween and incubated at 4 °C under constant agitation for 24 h. Then, the membranes were rinsed three times with TBS-Tween buffer and incubated overnight at 4 °C under constant agitation with the respective horseradish peroxidase-labeled antibody, diluted 1:10,000 in TBS-Tween. The secondary antibodies used were goat anti-rabbit IgG for all target proteins and goat anti-mouse IgG for GAPDH (ab97051 and ab205719, respectively, from Abcam PLC, Cambridge, Cambs, UK). To maximize sample efficiency, we used the same PVDF membrane (for the control group and MetS group, separately) to detect the target proteins. The membranes were stripped using a stripping buffer (glycine 25 mM, 0.01% SDS at pH 2) under constant shaking for 30 min at room temperature. Subsequently, the membranes were washed four times for 10 min each with Tris-Buffered Saline (Tris 20 mM, NaCl 0.16 M, Tween 20 0.001%, at pH 7.5). Every blot was treated with GAPDH antibody (sc-365062 from Santa Cruz Biotechnology, Santa Cruz, CA, USA) as a control. The chemiluminescent signal was developed using the Immobilon Western Substrate (Millipore Corporation, Billerica, MA, USA) and captured with a ChemiDoc™ Touch Imaging System (Bio-Rad Laboratories, Hercules, CA, USA). Digital images were densitometrically quantified utilizing Image Lab™ Software (Bio-Rad, Hercules, CA, USA), applying standardized background subtraction to each individual lane. The expected molecular weights and observed band sizes for the analyzed proteins were: TLR4 (~95 kDa), phospho-p65 NF-κB (~65 kDa), xCT (~55 kDa), GPX4 (~22 kDa), and GAPDH (~37 kDa). Protein normalization was executed by calculating the direct optical density ratio of each target protein band relative to its corresponding GAPDH loading control band within the same multiplexed or re-probed membrane. Regarding the inflammatory transcription factor, phosphorylated p65 (Ser536) was strictly normalized to GAPDH alone rather than total p65. Under the metabolic stress and chronic lipotoxic conditions characterizing the MetS lung phenotype, total p65 expression can undergo profound independent fluctuations in synthesis and turnover. Therefore, normalizing the active, phosphorylated state directly to a highly stable structural housekeeping protein like GAPDH ensures a reliable assessment of the absolute net concentration and tissue availability of the transcriptionally active factor per unit of total cellular protein, preventing artifacts associated with an unstable total p65.

### 2.10. Statistical Analysis

The data are presented as the mean ± standard error of the mean (SEM). Statistical analysis was performed using SigmaPlot software (version 11.0; Jandel Scientific, Corte Madera, CA, USA). Prior to analysis, the parametric assumptions of normality and homoscedasticity were verified using the Shapiro–Wilk and Levene’s tests, respectively. Data were analyzed utilizing a two-way Analysis of Variance (ANOVA) with metabolic status (MetS) and natural compound treatment (combined R + Q) as the main factors to evaluate their main effects and potential interactions. Upon finding statistically significant interaction terms, Tukey’s post-hoc test was applied for multiple pairwise comparisons. Statistical significance was strictly established at *p* < 0.05.

## 3. Results

### 3.1. Effect of Combined R + Q Administration in the MetS Rat Model

**Figure 2:**
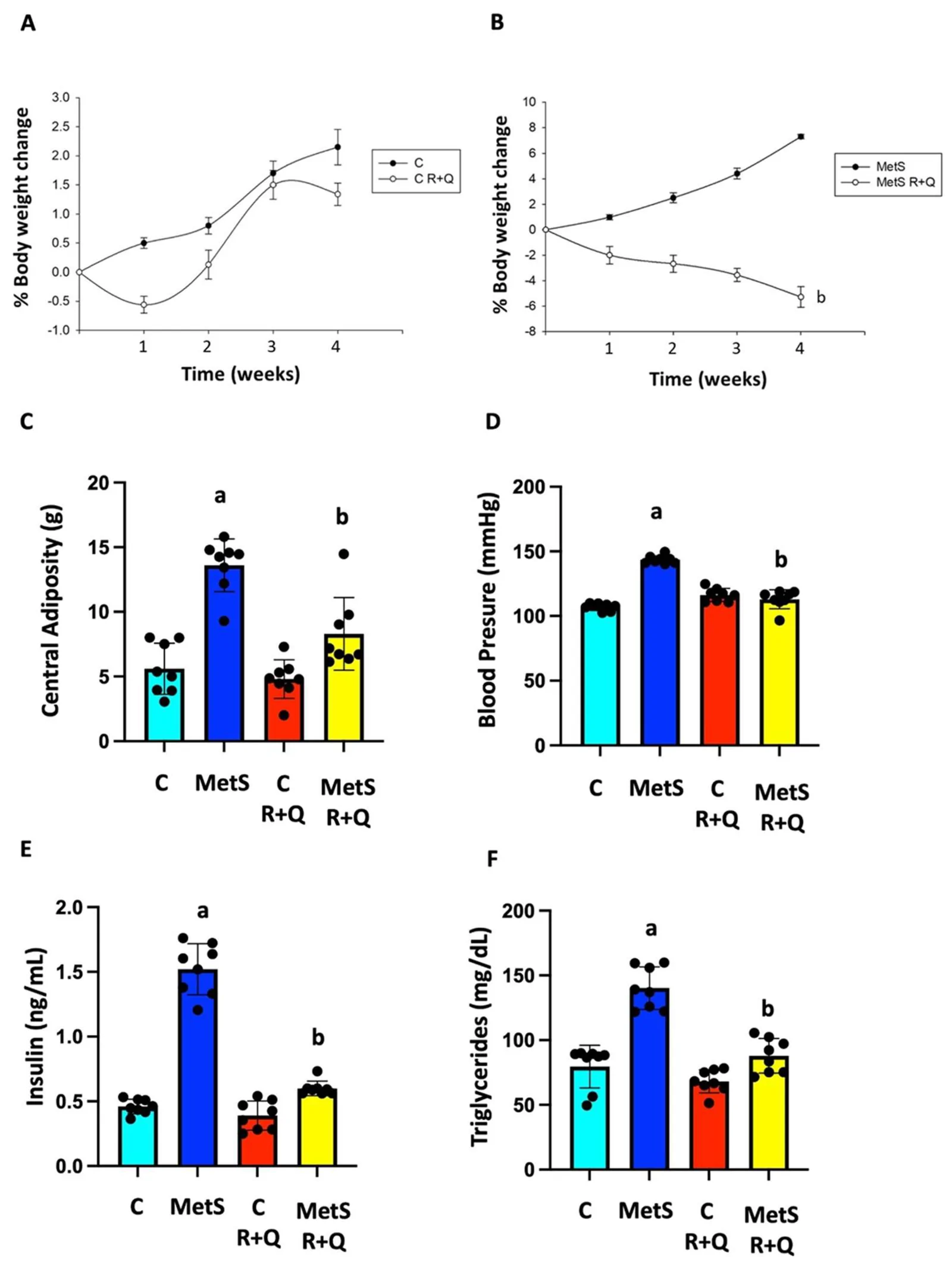
Supplementation with combined R + Q was effective in decreasing the signs of MetS. Changes in body weight over time in Control (**A**) and MetS (**B**) rats; all animals were weighed weekly during the 4-week intervention period. Visceral fat accumulation (**C**), systolic blood pressure (**D**), serum insulin levels (**E**), and serum triglyceride concentrations (**F**) were determined at the end of the treatment period (week 4). Data are expressed as mean ± SEM (*n* = 8 independent biological replicates per group). Statistical analysis was performed using a two-way ANOVA followed by Tukey’s post-hoc test. For body weight change, the analysis demonstrated a significant main effect of MetS (F_(1,28)_ = 9.68, *p* = 0.0042) without a significant treatment effect (*p* = 0.085) or interaction (*p* = 0.445). Conversely, highly significant MetS × treatment interactions were confirmed for visceral fat amount (F_(1,28)_ = 8.91, *p* = 0.0058), systolic blood pressure (F_(1,28)_ = 39.51, *p* < 0.0001), and triglyceride concentration (F_(1,28)_ = 16.65, *p* = 0.0003). ^a^ *p* < 0.01 vs. Control (C) without treatment; ^b^ *p* < 0.01 vs. MetS without treatment.

Our results show that chronic sucrose consumption induced a MetS phenotype characterized by increased body weight, central fat storage, elevated blood pressure, insulin, and triglyceride levels (Figure 2). Moreover, MetS rats had dyslipidemia (elevated non-HDL-C and reduced HDL-C) and developed IR (as measured by HOMA-IR). Fasting serum total cholesterol and glucose levels did not differ significantly among the groups (Table 1). In MetS rats, treatment with R + Q gradually prevented the increase in body weight, whereas this parameter remained constant in the C group (Figure 2A,B). Two-way ANOVA for final body weight change demonstrated a significant main effect of MetS (F_(1,28)_ = 9.68, *p* = 0.0042). In contrast, no significant main effect of treatment (F_(1,28)_ = 3.19, *p* = 0.085) or MetS x treatment interaction (F_(1,28)_ = 0.60, *p* = 0.445) was observed. The supplementation with natural compounds significantly decreased central adiposity and systolic arterial pressure, reversed dyslipidemia, and brought HOMA-IR levels back to normal in the MetS animals (Figure 2 and Table 1). Two-way ANOVA confirmed highly significant MetS × treatment interactions for visceral fat amount (F_(1,28)_ = 8.91, *p* = 0.0058), systolic blood pressure (F_(1,28)_ = 39.51, *p* < 0.0001), triglyceride concentration (F_(1,28)_ = 16.65, *p* = 0.0003), non-HDL cholesterol (F_(1,28)_ = 10.37, *p* = 0.0033), and the HOMA index (F_(1,28)_ = 10.37, *p* = 0.0033), mathematically verifying that the therapeutic efficacy of combined R + Q was strictly modulated by the baseline MetS phenotype. Tukey’s post hoc multiple-comparison tests further demonstrated that while MetS animals developed severe cardiometabolic alterations compared with controls (*p* < 0.01), combined R + Q administration robustly reversed hypertriglyceridemia, non-HDL lipid accumulation, and IR, successfully restoring all homeostatic parameters back to control baseline parameters (*p* > 0.05). Conversely, no significant variations or adverse physiological drops were triggered by the natural compounds within the control group (*p* > 0.30). Collectively, these results confirm the successful induction of MetS and establish a systemic lipid milieu that may predispose peripheral tissues, particularly the lung, to lipid accumulation and contribute to tissue injury.

### 3.2. Pulmonary Lipid Accumulation Is Prevented by Combined R + Q Supplementation in MetS Rats

**Figure 3:**
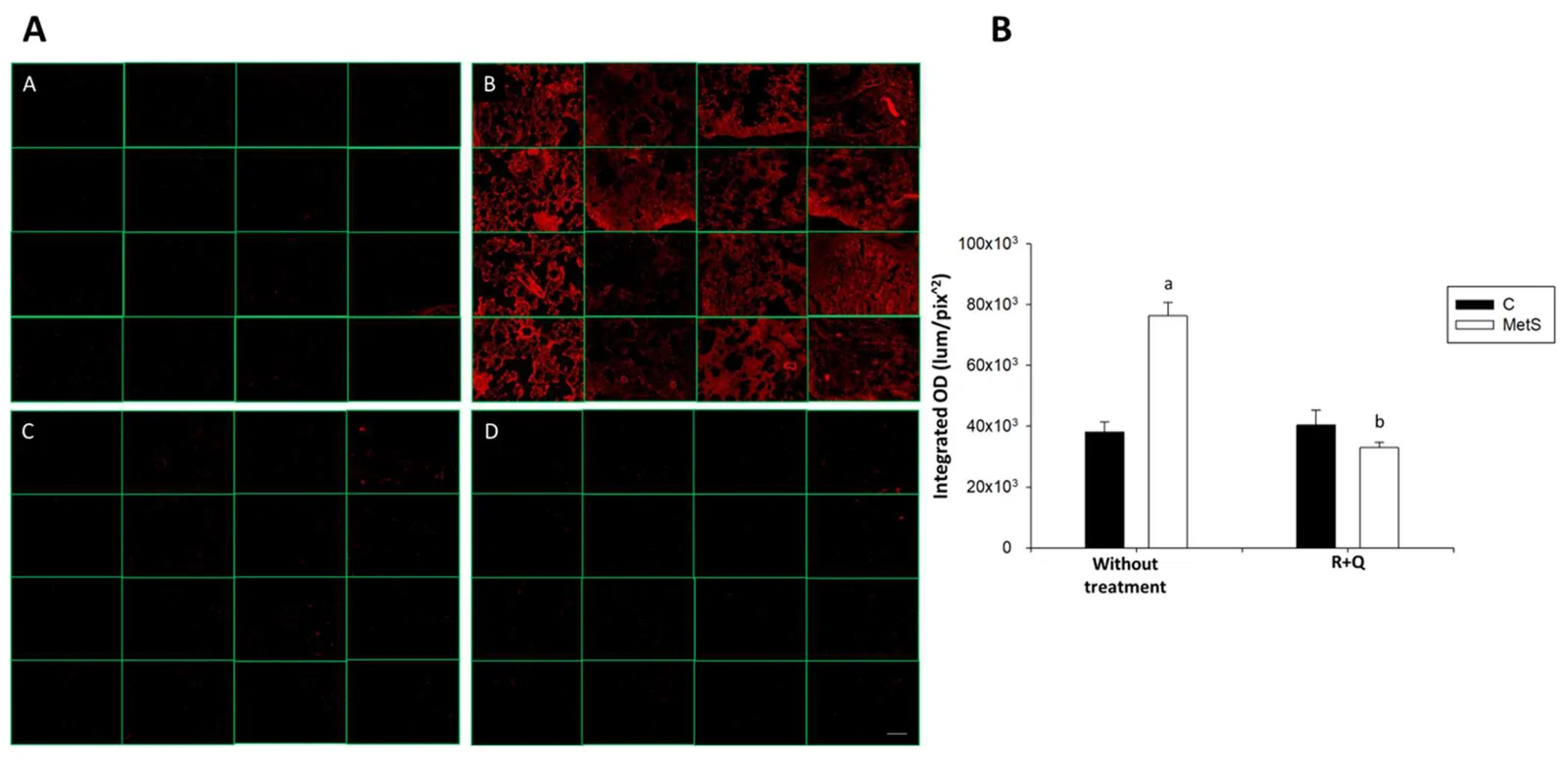
Dietary supplementation with the resveratrol + quercetin (combined R + Q) mixture prevents fat accumulation in the lungs of rats with metabolic syndrome (MetS). The proportions of neutral lipids, triglycerides, and cholesterol esters in the lungs of control and MetS rats were evaluated using the Oil Red (OR) assay and fluorescence imaging. (**A**) Representative fluorescence microphotographs of the experimental groups: Panel (**A**) = Control; Panel (**B**) = MetS; Panel (**C**) = Control plus combined R + Q treatment; Panel (**D**) = MetS plus combined R + Q treatment. The four representative images in each column correspond to different microscopic fields of the same tissue section from the same animal. (**B**) Densitometric quantification of OR label intensity presented as integrated optical density (IOD, lum/pix^2^). Data are expressed as mean ± SEM (*n* = 4) independent biological replicates per group. To avoid pseudoreplication, at least 16 microscopic fields across two independent tissue sections were quantified and averaged within each individual animal to yield a single statistical unit prior to analysis. Statistical analysis was performed using a two-way ANOVA followed by Tukey’s post-hoc test. ^a^ *p* < 0.01 vs. Control without treatment (Panel (**A**)); ^b^ *p* < 0.01 vs. MetS without treatment (Panel (**B**)). Scale bar = 50 µm.

Given elevated serum triglyceride levels, which are strongly associated with ectopic lipid accumulation in non-adipose organs, we evaluated the presence of fat deposits in the lungs across all experimental groups. Neutral lipids were detected by staining with OR in cross-sections of accessory lung from C and MetS rats, without treatment and with dietary supplementation of Q + R. The proportion of lipids in the alveolar walls of the lungs of MetS rats was twice that found in C animals. Importantly, supplementation with the combined R + Q mixture significantly reduced (52%) lipid deposition in MetS animals (Figure 3D), as evidenced by decreased OR staining intensity compared with untreated MetS rats (*p* < 0.001). No significant differences were observed between control and control + combined R + Q groups (Figure 3C), indicating that polyphenol supplementation did not alter basal pulmonary lipid content.

Two-way ANOVA for Oil Red O staining revealed a highly significant MetS × treatment interaction (F_(1,12)_ = 11.23, *p* = 0.0058). Tukey’s post-hoc analysis indicated that combined R + Q treatment successfully reversed the MetS-induced lipid accumulation (*p* = 0.0028), achieving a full normalization back to control levels (*p* = 0.8140) without affecting healthy controls (*p* = 0.9840). Collectively, these findings demonstrate that MetS promotes pulmonary lipid accumulation, while combined R + Q supplementation effectively prevents it, potentially limiting lipotoxicity that could otherwise cause inflammation, cell death, and tissue injury.

### 3.3. Combined R + Q Administration Decreases the Activation of the TLR4/NF-κB Signaling Pathway, Which Is Associated with Lung Fat Deposition

**Figure 4:**
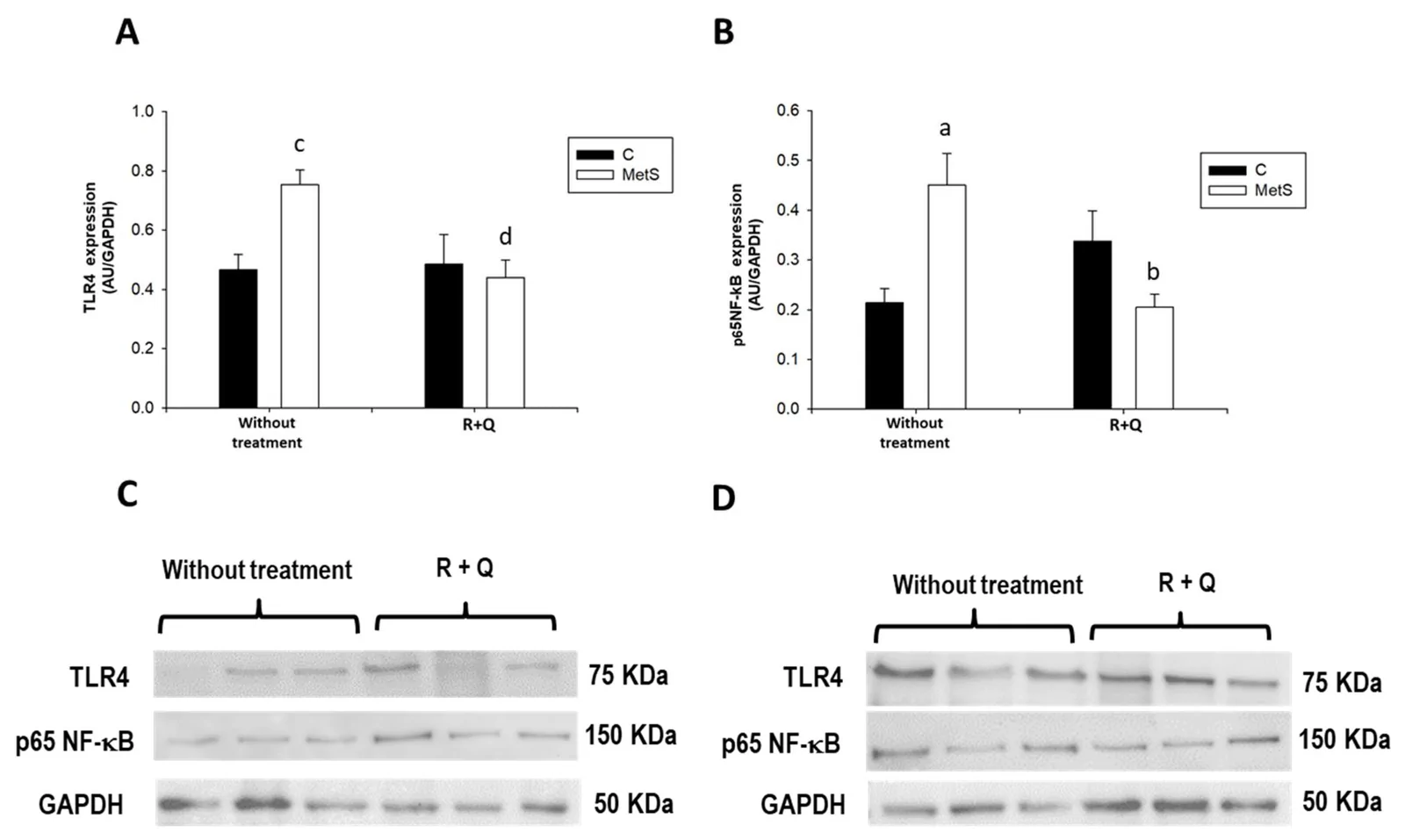
Resveratrol and quercetin (combined R + Q) supplementation downregulates TLR4 and NF-κB expression in the lungs of metabolic syndrome (MetS) rats. Expression levels were evaluated by Western blot in lung extracts from all experimental groups. (**A**) TLR4 protein expression and (**B**) phosphorylated p65 (p-p65) NF-κB expression. Representative Western blot bands from control (**C**) and MetS rats (**D**) are displayed. Data represent mean ± SEM (*n* = 3–6 independent biological replicates per group). Statistical analysis was performed using a two-way ANOVA followed by Tukey’s post-hoc test. For TLR4 expression, the analysis revealed a significant MetS × treatment interaction (F_(1,15)_ = 6.34, *p* = 0.0237). For p-p65 expression, a highly significant MetS × treatment interaction was observed (F_(1,20)_ = 17.44, *p* = 0.0005). ^a^ *p* < 0.01 vs. Control without treatment; ^b^ *p* < 0.01 vs. MetS without treatment; ^c^ *p* < 0.05 vs. Control without treatment; ^d^ *p* < 0.05 vs. MetS without treatment.

It has been reported that TLR4 is a key molecular bridge connecting lipid overload to inflammation; thus, we analyzed the expression levels of TLR4 and NF-κB in lung extracts. Two-way ANOVA for TLR4 expression revealed a significant MetS × treatment interaction (F_(1,15)_ = 6.34, *p* = 0.0237). Tukey’s post-hoc analysis indicated that TLR4 protein expression was significantly higher in MetS rats compared with controls, whereas the combined R + Q treatment successfully reversed this MetS-induced increase (*p* = 0.0327; Figure 4A), exerting no impact on the control group (*p* > 0.90). To evaluate the downstream activation of this inflammatory response, we measured the levels of phosphorylated p65 (p-p65) at Ser^536^ relative to GAPDH. Two-way ANOVA for p-p65 revealed a highly significant MetS × treatment interaction (F_(1,20)_ = 17.44, *p* = 0.0005), despite the absence of significant main effects for MetS (*p* = 0.479) or treatment (*p* = 0.329). Post-hoc testing demonstrated that MetS animals presented a distinct elevation of p-p65 compared with controls (*p* = 0.0121; Figure 4B), suggesting a clear trend toward enhanced transcriptionally active p65. Prominently, the combined R + Q treatment significantly suppressed this inflammatory response in the MetS group (*p* = 0.0078), successfully restoring p-p65 to near-baseline values without affecting control animals (*p* > 0.90). However, because total p65 levels were not determined, these data should be interpreted specifically as an elevation of this phosphorylated state rather than definitive whole-pathway activation, reflecting a localized modulation of this transcription factor.

### 3.4. MetS Promotes Pulmonary Iron Accumulation, Prevented by Combined R + Q Supplementation

**Figure 5:**
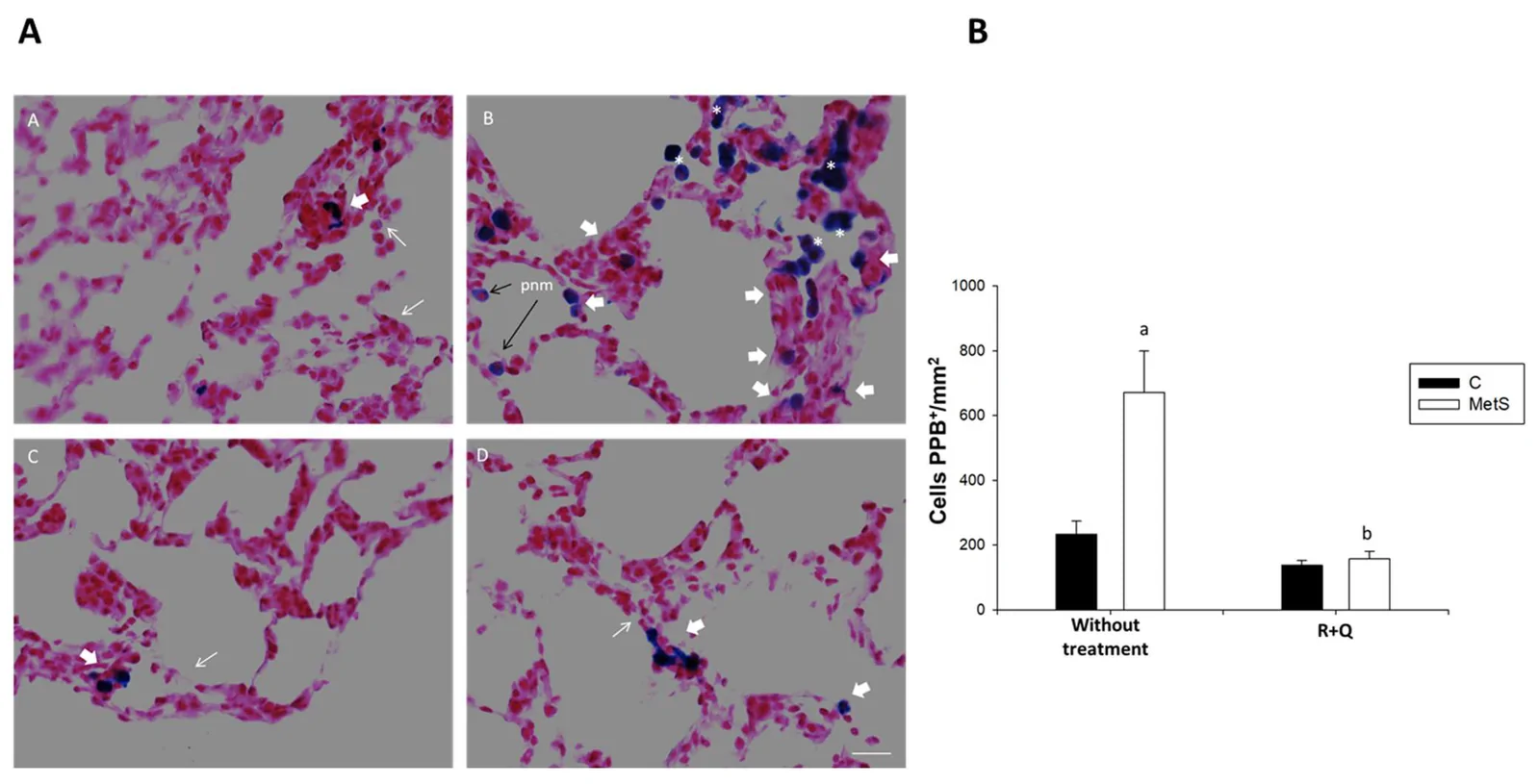
The accumulation of iron salts and hemosiderin in the lung cells from metabolic syndrome (MetS) rats was reversed by the administration of a resveratrol + quercetin combination (R + Q). Perls’ Prussian blue (PPB) staining was performed; most nuclei and the pulmonary parenchyma stained pink, contrasting with the blue coloration of iron deposits rather than other naturally occurring dark pigments in the lung. The number of PPB-positive cells was quantified in cross-sections of the accessory lung lobe from all experimental groups. (**A**) Representative optical microscopy images of the experimental groups: Panel (**A**) = Control; Panel (**B**) = MetS; Panel (**C**) = Control plus combined R + Q treatment; Panel (**D**) = MetS plus combined R + Q treatment. Thin white arrows indicate the inner wall of the alveoli, thin black arrows indicate PPB^+^ pneumocytes (pnm), thick white arrows indicate macrophages in the alveolar wall, and asterisks correspond to macrophages located in the alveolar lumen. Scale bar = 50 µm. (**B**) Quantitative analysis of the number of PPB^+^ cells normalized by tissue area (cells/mm^2^). Data are expressed as mean ± SEM (*n* = 4) independent biological replicates per group. To avoid pseudoreplication, multiple microscopic fields (4×) were fully stitched to reconstruct whole lung sections, yielding at least 16 total counts per condition that were averaged within each individual animal to establish a single statistical unit prior to evaluation. Statistical analysis was performed using a two-way ANOVA followed by Tukey’s post-hoc test. The analysis revealed a highly significant MetS × treatment interaction F_(1,12)_ = 13.75, *p* = 0.0030). ^a^ *p* < 0.01 vs. Control without treatment (Panel (**A**)); ^b^ *p* < 0.01 vs. MetS group without treatment (Panel (**B**)).

To further investigate whether lipotoxicity promotes iron accumulation, which in conjunction with oxidative stress, could lead to ferroptosis susceptibility. We evaluated iron salts and hemosiderin deposition in cross-sections of accessory lung tissue from all experimental groups using Perls’ Prussian blue staining. Our results indicate that the PPB^+^ stain was higher in the lungs of animals with MetS (87.59%) compared with the C group. The stain was localized in macrophages in the walls and alveolar spaces, and in some pneumocytes (Figure 5B). Two-way ANOVA for Perls’ Prussian Blue staining revealed a significant MetS × treatment interaction (F_(1,12)_ = 13.75, *p* = 0.0030). Tukey’s post-hoc analysis indicated that the MetS group exhibited a substantial increase in iron deposition compared with healthy controls (*p* = 0.0010). Quantitative analysis confirmed that the combined R + Q treatment significantly reduced iron deposition in MetS lungs to levels comparable to controls, while the healthy control group remained unchanged (*p* = 0.8607; Figure 5A,C).

In summary, these outcomes demonstrate that MetS disrupts lung iron homeostasis, and that this pathological accumulation is effectively prevented by the combined R + Q treatment.

### 3.5. MetS Induces Compensatory Upregulation of GPX4, Modulated by Combined R + Q Supplementation

**Figure 6:**
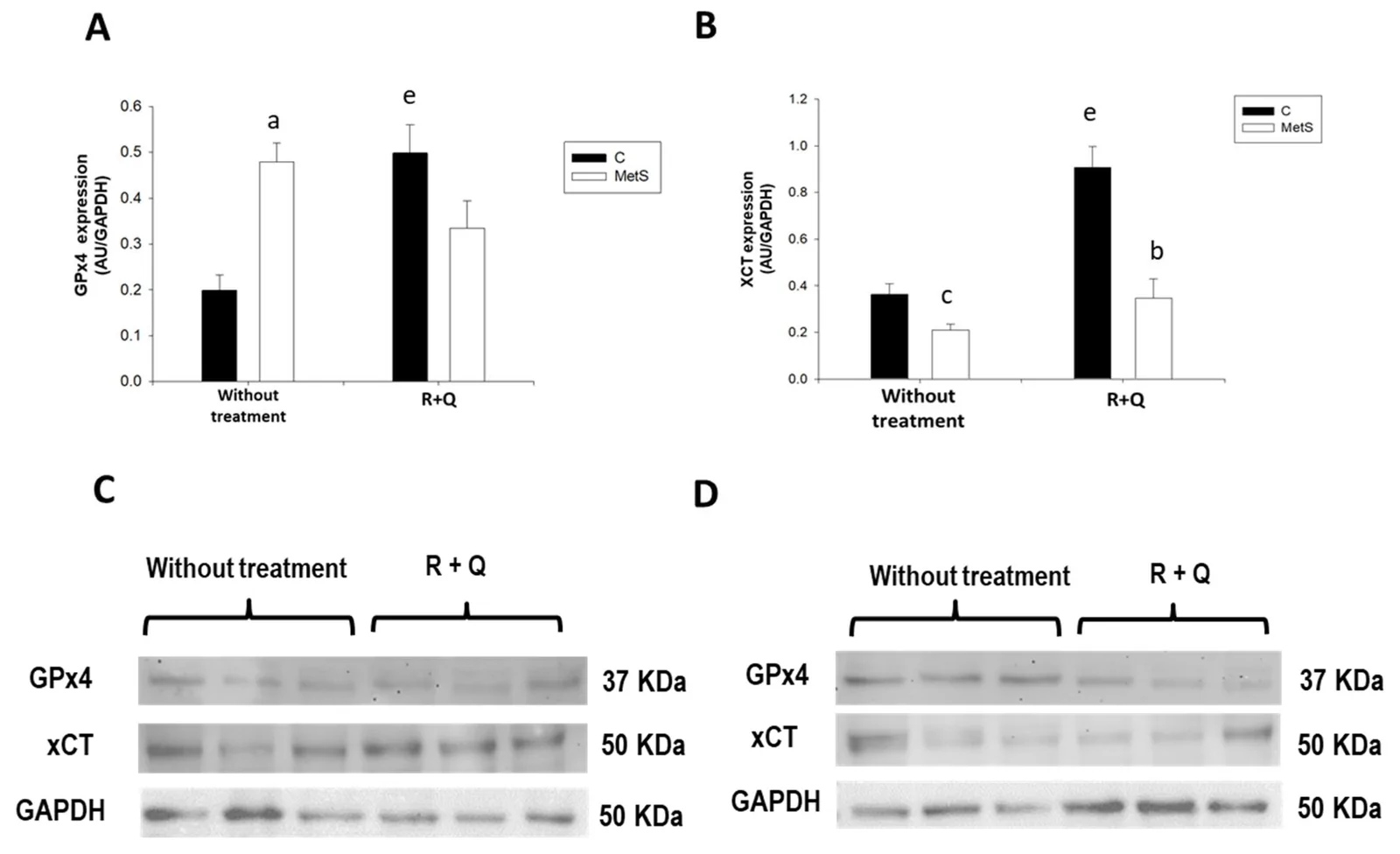
Effect of combined R + Q administration on GPX4 and xCT expression in lungs from Control and metabolic syndrome (MetS) rats. Expression levels of key ferroptosis-regulatory defenses were evaluated by Western blot in lung extracts from all experimental groups. (**A**) GPX4 protein expression; (**B**) xCT protein expression; (**C**) representative immunoblot images from the Control groups; (**D**) representative immunoblot images from the MetS groups. This figure shares the same internal GAPDH control as Figure 4. Data represent mean ± SEM (*n* = 3–6 independent biological replicates per group). Statistical analysis was performed using a two-way ANOVA followed by Tukey’s post-hoc test. For GPX4 expression, the analysis revealed a highly significant MetS × treatment interaction (F_(1,18)_ = 19.63, *p* = 0.0003); ^a^ *p* < 0.01 vs. Control without treatment; ^e^ *p* < 0.01 vs. Control without treatment. For xCT expression, a significant MetS × treatment interaction was similarly observed (F_(1,17)_ = 7.99, *p* = 0.0116; ^b^ *p* < 0.01 vs. MetS group without treatment; ^c^ *p* < 0.05 vs. Control without treatment; ^e^ *p* < 0.01 vs. Control without treatment).

To evaluate whether pulmonary lipid accumulation, TLR4/NF-κB signaling pathway, and iron deposition were associated with alterations in ferroptosis-regulatory defenses, GPX4 and xCT protein expressions were analyzed by Western blot (Figure 6). Two-way ANOVA for GPX4 expression revealed a significant MetS × treatment interaction (F_(1,18)_ = 19.63, *p* = 0.0003). Tukey’s post-hoc test showed that GPX4 expression was markedly increased in the lungs of MetS rats compared with controls (*p* = 0.0028), suggesting the activation of a compensatory antioxidant response under chronic metabolic stress. Given the concomitant lipid accumulation and iron deposition observed in MetS lungs, these elevated GPX4 levels likely reflect an increased demand for the detoxification of lipid hydroperoxides. Interestingly, while the combined R + Q treatment significantly increased GPX4 expression in the healthy control group (*p* = 0.0015), it did not induce further significant modifications in MetS animals (*p* = 0.248; Figure 6A).

Regarding xCT expression, two-way ANOVA revealed a significant MetS × treatment interaction (F_(1,17)_ = 7.99, *p* = 0.0116). Tukey’s post-hoc analysis indicated that while the combined R + Q treatment significantly enhanced xCT expression in controls (*p* < 0.001), it also acted as a potent upregulator in the MetS group, successfully rescuing its expression (Figure 6B). Collectively, these findings suggest that while the cystine/glutathione axis and GPX4 expression are robustly modulated by polyphenol supplementation, the adaptive response in MetS lungs involves a coordinated upregulation of both components to cope with lipid oxidative stress, which stabilizes and optimizes cellular defense systems following the combined R + Q treatment.

## 4. Discussion

MetS is a cluster of interrelated disorders, including central obesity, high blood pressure, IR, and dyslipidemia; while its cardiovascular consequences are well documented, pulmonary lipid remodeling has received considerably less attention. Lately, the association between MetS and pulmonary function has been reported [39,40]; several mechanisms, such as adiposity, IR, and fat-induced inflammation, have been proposed to explain this association, but they are not yet fully understood [2]. The principal findings of the present study were that: (i) chronic sucrose-induced MetS profoundly alters pulmonary lipid and iron homeostasis; (ii) chronic sucrose-induced MetS activates the TLR4/NF-κB signaling pathway, promoting inflammatory responses; (iii) additionally, this condition induces lipotoxicity, inflammation, and iron accumulation, establishing a tissue microenvironment consistent with increased susceptibility to ferroptosis; (iv) combined R + Q supplementation effectively prevented lipid accumulation, reduced protein expression of TLR4, and NF-κB, iron deposition, as well as modulated ferroptosis-regulatory protein expression in lung tissue. It can therefore be concluded that the polyphenol mix had a protective effect against pulmonary damage associated with MetS.

The results in Figure 2 and Table 1 confirm the development of MetS following chronic sucrose exposure; the animals exhibit increased central adiposity, hypertension, dyslipidemia, hyperinsulinemia, and IR. Besides, IR is associated with uncontrolled lipolysis, releasing free fatty acids that increase serum levels and eventually result in ectopic fat deposition. This is detrimental because it leads to lipotoxicity, in which the accumulation of lipid metabolic intermediates in organs can alter metabolic processes and impair organ function [41,42,43]. These ectopic fat deposits generate high inflammatory and endocrine/paracrine activity, contributing to low-grade systemic inflammation [44,45], which correlates with hypoxia, macrophage infiltration, and chronic low-grade systemic inflammation, generating systemic metabolic stress [43] in different organs and tissues [34,41,42,46], such as lungs [47]. Our findings reveal significant accumulation of neutral lipids in the lungs of MetS animals (Figure 3), suggesting that chronic systemic metabolic imbalance extends beyond classical metabolic organs. These results are consistent with previous reports in animal models that have demonstrated increased lipid droplet formation in alveolar epithelial cells and macrophages under metabolic stress [48]. In addition, fat deposition damages the alveolar ultrastructure, reduces surfactant production, and alters lipid composition, thereby promoting lung tissue fibrosis and leading to pulmonary dysfunction [49]. Moreover, the observed lipid accumulation in MetS lungs is consistent with the systemic dyslipidemia described in Table 1 and suggests increased availability of oxidizable lipid substrates within pulmonary tissue. In this regard, it has been reported that cells in the lung can take up fatty acids from extracellular sources, including lipoproteins (chylomicrons, LDL, and VLDL) and free fatty acids obtained through macrophage phagocytosis; all of these play a crucial role in maintaining the intracellular lipid pool [9].

Furthermore, it is well known that ectopic lipid deposition enhances susceptibility to oxidative stress by increasing the availability of polyunsaturated fatty acids, which are highly vulnerable to peroxidation [30]. Therefore, pulmonary lipid accumulation in MetS rats might constitute a primary metabolic event that sensitizes lung tissue to redox imbalance.

As previously reported, supplementation with combined R + Q was effective in reducing signs of our MetS rat model; however, the important finding in this study is that natural compounds significantly diminished lipid deposition in the lungs of MetS rats. Previous studies from our group and others have demonstrated that modulation of lipid metabolism and reduction in lipotoxicity mitigate ectopic lipid deposition and its associated tissue damage, particularly in the liver, skeletal muscle, and kidney [36,50,51]. However, the present study addresses a significant knowledge gap. While the beneficial effects of R and Q have been widely attributed to their antioxidant and anti-inflammatory properties, most studies have focused on preventing lipotoxicity or attenuating LPS-induced lung inflammation. By contrast, their effects on ectopic fat deposition and its contribution to lung metabolic remodeling/alterations in MetS remain poorly understood.

On the other hand, several studies have reported associations between metabolic dysfunction and various respiratory conditions, including asthma, chronic obstructive pulmonary disease (COPD), interstitial lung disease, obstructive sleep apnea, and pulmonary hypertension [45]; and that iron is of fundamental importance in mediating oxidative lung injury and the pathogenesis of COPD by generating toxic hydroxyl radicals and promoting intracellular bacterial growth. Most of the iron in the respiratory tract, including excess iron, is stored within alveolar macrophages and airway epithelial cells in insoluble hemosiderin-laden macrophages (HLMs) [50].

In the present study, we found that lung tissue from rats with MetS presents approximately three times more HLMs compared with the control group (Figure 5A,B), while lung tissue from animals with MetS supplemented with combined R + Q showed a proportion of HLMs equivalent to those in the C group (Figure 5D). Under physiological conditions, pulmonary iron homeostasis is tightly controlled to prevent tissue toxicity and oxidative damage. The presence of hemosiderin aggregates inside alveolar macrophages is a well-documented pathological sign of altered iron processing; however, its precise triggering mechanism in this model remains to be fully elucidated. Given that our current experimental design did not directly measure pulmonary arterial pressure, endothelial permeability, or vascular leakage markers, the presence of these hemosiderin-laden cells should be interpreted cautiously as a plausible physiological hypothesis rather than a definitive, demonstrated mechanism. Mechanistically, it could be hypothesized that chronic systemic inflammation, oxidative stress, and metabolic distress associated with MetS promote a low-grade, localized endothelial dysfunction within the pulmonary microvasculature. This alteration could lead to subtle, chronic damage to blood vessels with low-grade microhemorrhages. In response to this potential tissue injury, macrophages may migrate to the site and phagocytize red blood cells, which are subsequently degraded—alongside hemoglobin and hematin—releasing free iron that utilizes ferritin as an intermediary to accumulate as hemosiderin Mechanistically, it could be hypothesized that chronic systemic inflammation, oxidative stress, and metabolic distress associated with MetS promote a low-grade, localized endothelial dysfunction within the pulmonary microvasculature. This alteration could lead to subtle, chronic damage to blood vessels with low-grade microhemorrhages. In response to this potential tissue injury, macrophages may migrate to the site and phagocytize red blood cells, which are subsequently degraded—alongside hemoglobin and hematin—releasing free iron that utilizes ferritin as an intermediary to accumulate as hemosiderin [51]. While the precise molecular mechanisms by which the combined R + Q treatment modulates lipid accumulation and HLMs in the lung under MetS conditions require further clarification, polyphenols are well documented to possess potent antioxidant, anti-inflammatory, and iron-modulating properties [42,43]. In pulmonary models, R has been shown to attenuate oxidative stress and inflammatory injury [45], while Q improves mitochondrial function and reduces lipid peroxidation [46]. Therefore, this distinct iron accumulation pattern serves as a descriptive marker of altered pulmonary iron homeostasis and localized tissue stress, and the combined R + Q supplementation successfully orchestrates a full therapeutic stabilization of this axis by potentially preserving microvascular integrity and mitigating the underlying chronic inflammatory tone.

Fatty acids, specifically saturated fatty acids, are endogenous ligands of TLR4, a critical sensor in lung parenchymal cells that recognizes pathogens (such as LPS) and damage-associated molecular patterns (DAMPs), triggering significant proinflammatory responses via NF-κB activation. Much evidence suggests that the TLR4/NF-κB signaling pathway is a key mediator in the link between obesity and pulmonary dysfunction, because it causes fibrosis, chronic inflammation, and acute lung injury [52,53]. In this regard, Figure 4 shows that TLR4 and phosphorylated p65 NF-κB protein levels are higher in lungs from MetS rats than in control animals. In the context of obesity and MetS, TLR4 expression increases, which is associated with IR. TLR4 expression is increased in obesity in most studies. Goldklang et al. [54] reported that lungs from high-fat-diet mice showed increased expression and subsequent activation of the TLR4 pathway; likewise, obese individuals exhibit greater TLR4 expression, which can worsen or increase susceptibility to lung diseases [55]. Our findings in Figure 4 demonstrated that the administration of combined R + Q mix significantly decreased TLR4 and phosphorylated p65-NF-κB protein expression levels in lungs from MetS rats. These results are consistent with reports on the protective effect of R or Q against lung diseases by inhibiting the TLR4 signaling pathway [56,57]. Although the lack of total p65 evaluation prevented calculating the p-p65/total p65 ratio, phosphorylation at Ser536 independently enhances the transactivation potential of p65. Thus, while elevated p-p65 (Ser536) normalized to GAPDH supports a state of heightened inflammatory signaling, further quantification of total p65 and nuclear translocation is required to fully map NF-κB activation dynamics. Importantly, a key strength of our study is the utilization of a polyphenol mixture, which highlights the therapeutic potential of combinatorial approaches in boosting protective outcomes.

**Figure 7:**
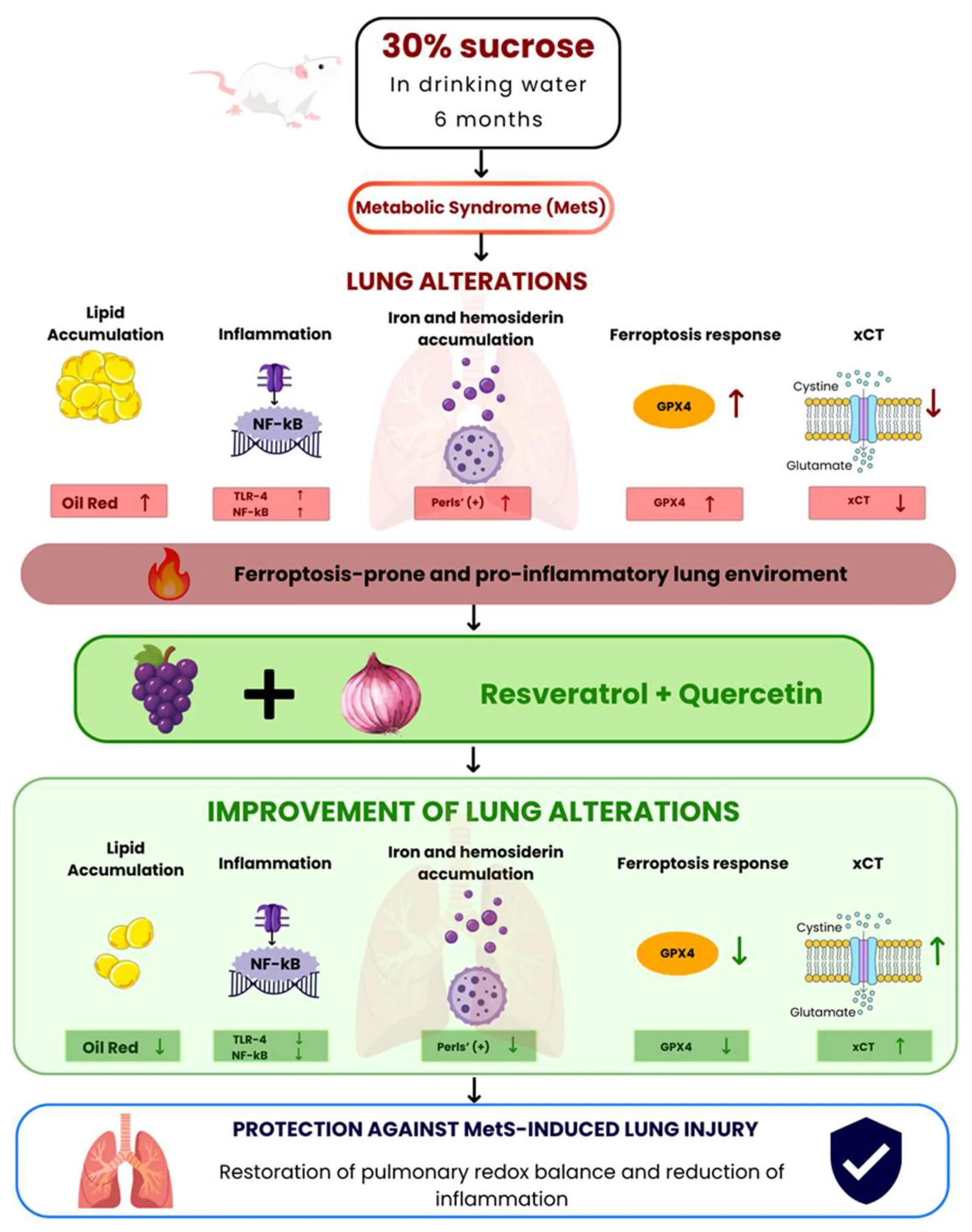
Pulmonary alterations induced by metabolic syndrome (MetS) and their modulation by resveratrol + quercetin (combined R + Q) supplementation. Chronic sucrose-induced MetS promoted pulmonary lipid accumulation, activation of the TLR4/NF-κB inflammatory signaling pathway, iron and hemosiderin deposition, and increased GPX4 expression, generating a pro-inflammatory and pro-oxidative lung environment associated with increased susceptibility to ferroptosis. Supplementation with combined R + Q reduced lipid deposition, inflammatory signaling, iron and hemosiderin accumulation, and GPX4 overexpression, suggesting restoration of pulmonary redox homeostasis and attenuation of metabolic lung injury. Upward arrows indicate an increase, and downward arrows indicate a decrease.

Importantly, an additional relevant finding of the present study was the increased expression of TLR4 and NF-κB in MetS lungs, suggesting activation of inflammatory signaling pathways under chronic metabolic stress. These observations are particularly relevant considering the emerging evidence linking ferroptosis-related pathways and inflammation as interconnected pathological processes. For instance, recent studies indicate that ferroptosis-related processes may be associated with inflammatory signaling cascades, especially those involving TLR4 and NF-κB activation [58]. Lipid peroxidation products and oxidized phospholipids generated during ferroptotic stress can function as DAMPs, promoting activation of innate immune pathways and amplifying inflammatory responses [59]. TLR4 is a critical sensor of metabolic and oxidative stress and has been implicated in obesity-associated inflammation and pulmonary injury [55]. Activation of TLR4 signaling induces NF-κB translocation, leading to transcription of pro-inflammatory mediators and reinforcement of oxidative stress pathways [60]. In turn, chronic NF-κB activation contributes to mitochondrial dysfunction, reactive oxygen species generation, and disruption of cellular redox homeostasis, thereby creating conditions that may further increase ferroptotic susceptibility [61]. The simultaneous increase in lipid accumulation, iron deposition, GPX4 upregulation, and activation of the TLR4/NF-κB axis observed in our study suggests a coordinated pro-oxidative and pro-inflammatory pulmonary microenvironment in MetS animals. These findings suggest the concept that ferroptosis-related metabolic remodeling and inflammatory signaling are likely not independent events but rather interconnected processes that may synergistically contribute to lung dysfunction during MetS, something that warrants further investigation. Importantly, supplementation with combined R + Q attenuated these alterations, consistent with previous evidence that polyphenols suppress TLR4/NF-κB signaling and restore redox homeostasis [62]. A critical mechanistic consideration is whether the observed attenuation of pulmonary lipid deposition, iron accumulation, and TLR4/NF-κB signaling reflects a direct action of these natural compounds within the lung or is a secondary consequence of systemic metabolic improvement. While the robust amelioration of central adiposity, hypertension, dyslipidemia, and insulin resistance undeniably alleviates overall organic stress, our two-way ANOVA findings reveal significant MetS treatment interactions specifically for pulmonary TLR4, NF-κB, GPX4, and xCT expressions. These interactions mathematically demonstrate that the efficacy of combined R + Q is strictly orchestrated by the local pathological microenvironment rather than operating as a passive secondary downstream effect of systemic recovery. Therefore, we do not rule out the secondary consequences of systemic metabolic improvement; rather, we propose that the pulmonary benefits of combined R + Q involve an intricate combination of localized, context-dependent bioactive effects within the lung tissue operating in tandem with the undeniable homeostatic advantages provided by systemic metabolic restoration. Finally, a key limitation of this work is that R and Q were not evaluated independently. This experimental design prevents us from determining if the outcomes stem from one specific compound or from a synergistic relationship between both. To clarify these interactive dynamics, future multi-arm studies including isolated R and Q groups are warranted to establish each component’s precise contribution within the combined treatment. Therefore, the protective effects observed with the combined R + Q treatment in this study may involve the simultaneous modulation of ferroptosis susceptibility and inflammatory signaling pathways (Figure 7).

The coexistence of lipid accumulation and iron deposition observed in our study (Figure 3B and Figure 5B) suggests a pulmonary environment that may favor ferroptosis susceptibility. Ferroptosis is characterized by iron-dependent accumulation of lipid peroxides and is tightly regulated by the GPX4/glutathione axis [63]. Although direct lipid peroxidation measurements were not performed, the combined lipid–iron remodeling detected here strongly supports vulnerability to ferroptotic signaling in MetS lungs.

One of the most notable findings of this study is the marked upregulation of GPX4 in MetS lungs (Figure 6A). GPX4 is the central enzyme responsible for detoxifying phospholipid hydroperoxides and preventing lipid peroxidation-associated cellular damage [23]. Increased GPX4 expression has been described as an adaptive response to sustained oxidative or metabolic stress [64]. In metabolic disease models, elevated GPX4 levels have been reported in tissues exposed to chronic lipid overload, suggesting a protective mechanism to maintain membrane integrity under lipotoxic conditions [65]. Rather than indicating active lipid peroxidation-associated cell death, elevated GPX4 in MetS animals likely reflects a compensatory mechanism to counterbalance persistent oxidative lipid stress. Interestingly, combined R + Q treatment increased GPX4 expression in healthy control rats. This finding should not be interpreted as evidence of oxidative or metabolic stress, since GPX4 is not only a regulator of ferroptosis but also an essential antioxidant enzyme that maintains membrane lipid homeostasis. Polyphenols such as R and Q have been shown to activate endogenous cytoprotective pathways, including Nrf2- and SIRT1-dependent signaling, thereby enhancing the expression of antioxidant enzymes under physiological conditions. Therefore, the increased GPX4 expression observed in the Control + combined R + Q group likely reflects an adaptive activation of endogenous antioxidant defenses rather than a disturbance of redox homeostasis. By contrast, although GPX4 expression was also elevated in MetS animals, this increase was accompanied by iron accumulation and reduced xCT expression, suggesting that GPX4 upregulation represents a compensatory but insufficient response to persistent oxidative stress when isolated. Since xCT is essential for cystine uptake and glutathione synthesis, its downregulation under MetS conditions may limit GPX4 antioxidant activity despite increased protein expression. Following combined R + Q treatment, the significant upregulation and restoration of xCT expression (*p* = 0.0116; Figure 6B), along with reduced iron accumulation and inflammatory burden, likely optimized the overall efficiency of the glutathione system. Interestingly, GPX4 expression stabilized and maintained its elevated plateau following R + Q treatment in MetS animals (*p* = 0.248). This lack of further overexpression, coupled with the robust rescue of xCT, indicates that the polyphenol combination successfully reinforced the tissue’s coordinated redox axis rather than inducing a standalone enzyme demand.

Interestingly, the biological activity of resveratrol is likely to reflect its pleiotropic effects on multiple cellular stress-response pathways rather than a single molecular target. In addition to its reported antioxidant and anti-inflammatory properties, resveratrol has been discussed in the context of natural-product modulation of DNA-damage response and checkpoint signaling, including the ATR–CHK1 pathway [66]. These broader signaling effects may contribute to the context-dependent cellular responses elicited by resveratrol under conditions of metabolic and oxidative stress. Moreover, polyphenols have been reported to restore redox equilibrium by reducing reactive oxygen species generation and improving mitochondrial bioenergetics [67]. Thus, our data support the concept that MetS establishes a pulmonary environment characterized by heightened susceptibility to ferroptosis, which adaptive antioxidant defense systems actively restrain. This defense mechanism may delay or prevent overt ferroptotic cell death despite lipid and iron overload. The significant modulation of xCT expression further supports the coordinated involvement of this redox axis (Figure 6B). Together, these findings suggest that MetS is associated with pulmonary alterations that may increase susceptibility to ferroptosis and may elicit compensatory changes in antioxidant and ferroptosis-regulatory defenses. R + Q treatment attenuated these alterations and was associated with a partial normalization of the evaluated ferroptosis-related protein profile.

The alterations observed in ferroptosis-regulatory proteins, together with pulmonary lipid accumulation and iron deposition, suggest an altered ferroptosis-regulatory state in MetS lungs. However, these findings should not be interpreted as direct evidence of ferroptotic cell death, since the present study did not assess key features of ferroptosis, including lipid peroxidation and ferroptosis-specific pharmacological rescue. Thus, our findings support a pulmonary environment potentially conducive to ferroptosis rather than establishing ferroptosis as a mechanism of lung injury.

Although the observed biochemical, histological, and molecular alterations are consistent with a pulmonary environment potentially conducive to ferroptosis, future studies incorporating lipid peroxide quantification and ferroptosis-specific modulators will be necessary to establish a direct mechanistic link. In addition, only male rats were included in the experimental design; therefore, whether these findings and the protective effects of R plus Q are reproducible in females remains to be determined. Although increased iron deposition in HLM was observed, quantitative discrimination between macrophages located in the alveolar walls and those present in the alveolar lumen was not performed, limiting a more detailed characterization of their distribution. Furthermore, the present study focused exclusively on the combined administration of R and Q; therefore, the individual contribution of each compound and the extent of their potential combinatorial or additive interactions warrant further investigation. Ideally, future studies with independent treatment groups (MetS + resveratrol alone and MetS + quercetin alone) will be needed to dissect these dynamics. Addressing these limitations will contribute to a more comprehensive understanding of the mechanisms underlying MetS-associated lung injury and the therapeutic potential of these polyphenols.

Our results highlight that the lung is increasingly recognized as a target of the systemic consequences of obesity and metabolic dysfunction, although several aspects of the underlying mechanisms remain to be elucidated. The combination of lipid remodeling, TLR4/NF-κB signaling pathway, and iron accumulation suggests that MetS creates a pulmonary environment that may favor ferroptosis. These alterations may contribute to increased vulnerability to secondary insults such as infection, environmental pollutants, or ischemic stress. Targeting lipid–iron and TLR4/NF-κB signaling pathway interactions may therefore represent a novel therapeutic strategy for preventing metabolic lung remodeling or alterations of metabolic origin.

## 5. Conclusions

Chronic sucrose-induced MetS is closely associated with pulmonary metabolic remodeling/alterations by promoting lipid accumulation, iron deposition, and compensatory upregulation of the antioxidant enzyme GPX4. These alterations are consistent with the establishment of a pro-inflammatory state via activation of the TLR4/NF-κB signaling pathway and a pulmonary environment that is probably conducive to ferroptosis. Combined R + Q supplementation may represent a promising strategy in attenuating pulmonary metabolic remodeling/alterations caused by metabolic dysfunction by downregulating ectopic fat deposition, TLR4/NF-κB signaling pathway, and iron accumulation.

## Figures and Tables

**Table 1:** The effects of combined R + Q administration on serum total cholesterol, HDL-c, non-HDL-c, glucose levels and HOMA-IR index from control and MetS rats.

	Control	Control Plus COMBINED R + Q	MetS	MetS Plus COMBINED R + Q
Total Cholesterol (mg/dL)	57.3 ± 4.8	59.3 ± 4.0	61.2 ± 2.8	57.6 ± 2.1
HDL-c (mg/dL)	28.1 ± 2.3	29.6 ± 1.9	17.8 ± 1.1 ^a^	22.4 ± 2.0 ^b^
Non-HDL-c (mg/dL)	21.9 ± 1.6	19.4 ± 0.9	36.9 ± 3.8 ^a^	19.6 ± 2.1 ^b^
Glucose (mg/dL)	101.0 ± 2.3	91.1 ± 3.8	92.8 ± 1.9	102.1 ± 1.9
HOMA-IR	1.2 ± 0.19	0.85 ± 0.09	2.3 ± 0.30 ^a^	0.79 ± 0.05 ^b^

Values are mean ± SEM. Abbreviations: HOMA-IR = homeostatic model assessment of insulin resistance; HDL-c: high-density lipoprotein cholesterol; resveratrol + quercetin (R + Q). *n* = 8 animals per group; ^a^ *p* < 0.01 vs. C; ^b^ *p* < 0.01 vs. MetS without treatment.

## Data Availability

The original contributions presented in this study are included in the article. Further inquiries can be directed to the corresponding author.
